# Influence of strategic points in the dispersion of *Aedes aegypti* in infested areas

**DOI:** 10.11606/S1518-8787.2019053000702

**Published:** 2019-03-27

**Authors:** Gerson Laurindo Barbosa, Mariana de Oliveira Lage, Valmir Roberto Andrade, Antônio Henrique Alves Gomes, Jose Alberto Quintanilha, Francisco Chiaravalloti-Neto

**Affiliations:** I Secretaria de Estado da Saúde. Superintendência de Controle de Endemias. São Paulo, SP, Brasil; II Universidade de São Paulo. Programa de Pós-Graduação em Ciências Ambientais. São Paulo, SP, Brasil; III Universidade de São Paulo. Escola Politécnica. Departamento de Engenharia de Transportes. São Paulo, SP, Brasil; IV Universidade de São Paulo. Faculdade de Saúde Pública. Departamento de Epidemiologia. São Paulo, SP, Brasil

**Keywords:** *Aedes aegypti*, growth & development, Oviposition, Spatial Analysis, Vector Control, Communicable Disease Control, *Aedes aegypti*, crescimento & desenvolvimento, Oviposição, Análise Espacial, Controle de Vetores, Controle de Doenças Transmissíveis

## Abstract

**OBJECTIVE:**

To evaluate whether sites with large amount of potential breeding sites for immature forms of *Aedes aegypti*, called strategic points, influence in the active vector's dispersion into properties in their surroundings.

**METHODS:**

We selected four areas in the municipality of Campinas, three of them with strategic points classified as high, moderate, and low risk according to infestation and a control area, without strategic points. Between October 2015 and September 2016, we monthly installed oviposition traps and evaluated the infestation by *Ae. aegypti* in all properties of each selected area. To verify if there was vector dispersion from each strategic point, based on its location, we investigated the formation of clusters with excess of eggs or larvae or pupae containers, using the *G*_i_ spatial statistics.

**RESULTS:**

The amount of eggs collected in the ovitraps and the number of positive containers for *Ae. aegypti* did not show clusters of high values concerning its distance from the strategic point. Both presented random distribution not spatially associated with the positioning of strategic points in the area.

**CONCLUSIONS:**

Strategic points are not confirmed as responsible for the vector's dispersion for properties in their surroundings. We highlight the importance of reviewing the current strategy of the vector control program in Brazil, seeking a balance from the technical, operational, and economic point of view, without disregarding the role of strategic points as major producers of mosquitoes and their importance in the dissemination of arboviruses in periods of transmission.

## RESUMO

**OBJETIVO:**

Avaliar se locais com grande quantidade de potenciais criadouros de formas imaturas de *Aedes aegypti*, denominados pontos estratégicos, influenciam a dispersão ativa do vetor aos imóveis no seu entorno.

**MÉTODOS:**

Foram selecionadas quatro áreas no município de Campinas, três delas com pontos estratégicos classificados como alto, médio e baixo risco segundo a infestação e uma área controle, sem ponto estratégico. Entre outubro de 2015 e setembro de 2016, instalaram-se mensalmente armadilhas de oviposição e avaliou-se a infestação por *Ae. aegypti* em todos os imóveis de cada área selecionada. Para verificar se houve dispersão do vetor a partir de cada ponto estratégico, com base em sua localização, investigou-se a formação de aglomerados com excesso de ovos ou de recipientes com larvas ou pupas, utilizando a estatística espacial *G*_i_.

**RESULTADOS:**

o número de ovos coletados nas ovitrampas e o número de recipientes positivos para *Ae. aegypti* não apresentaram aglomerados de altos valores relativos à sua distância do ponto estratégico. Ambos apresentaram distribuição aleatória não associada espacialmente com o posicionamento dos pontos estratégicos na área.

**CONCLUSÕES:**

Pontos estratégicos não se confirmaram como responsáveis pela dispersão do vetor para os imóveis no seu entorno. Destaca-se a importância de rever a estratégia atual do programa de controle de vetores do Brasil, buscando um equilíbrio do ponto de vista técnico, operacional e econômico, sem desconsiderar o papel dos pontos estratégicos como grandes produtores de mosquitos e sua importância na disseminação de arboviroses em momentos de transmissão.

## INTRODUCTION

*Aedes aegypti* is a predominantly urban vector, initially recognized as the causative agent of dengue epidemics in tropical and subtropical countries, affecting millions of people in recent decades, in addition to transmitting the yellow fever virus and the zika and chikungunya viruses, whose infections account for high incidence in Brazil since 2015^1^. Over 80% of the world's population is at risk of diseases transmitted by vectors^2^, many of which are concentrated in poor communities of the aforementioned regions.

Nowadays, dengue control is basically centered in combating the *Ae. aegypti* vector in its different stages. Vaccines are being developed, but there are still challenges concerning the immune response to all serotypes^3^ and, consequently, concerning its use in the routine of control programs. According to Achee et al.^4^, when an effective vaccine for dengue becomes available, public health programs will continue to depend on vector control, because both strategies complement and improve each other. Furthermore, there is no vaccine for zika and chikungunya, which corroborates the importance of the likely continuity of combating the mosquito, which must be technically and scientifically improved.

In Brazil, a series of activities for controlling *Ae. aegypti* are routinely developed, based on the *Programa Nacional de Controle da Dengue* (PNCD – National Dengue Control Program)^5^. One of the strategies seeks to prioritize buildings with large amount of potential breeding sites for the immature forms of the mosquito, which mainly consist in tires, water tanks, cans, bottles, and other objects that retain water. These properties are referred to as strategic points (SP) and have been a constant target of plans for intensifying the control of the vector, proposed for interepidemic periods^5^ ^,^ ^6^. Their features require the formation of specialized teams for periodic inspection, treatment, and monitoring. However, many municipalities do not meet the recommendations of PNCD, mainly regarding periodicity, the owner's involvement, effective health surveillance actions, and chemical treatment.

The importance of the SP would be supported because they present high productivity of mosquitoes and behave as vector dispersers to neighboring areas, generating, feeding, and keeping smaller outbreaks. The positivity of SP in the state of São Paulo is higher than the positivity found in residential properties, as measured by the Building Infestation Index (BII)^7^. The role of SP as dispersers of the vector was evidenced by a study carried out in São Paulo at the beginning of reinfestation by *Ae. aegypti* in the early 1980s, when the majority of detections (73%) primarily occurred in those points^8^ and then in residential buildings.

Although they have higher positivity than other types of properties, a question to be studied is whether SP have a role in the vector's dispersion in neighboring areas. The lack of research on this issue can be considered a gap in the knowledge about the behavior of *Ae. aegypti*.

Thus, our objectives were to evaluate, in areas with a history of vector infestation, the level of the SP infestation and of properties in their surroundings, and also to verify whether they have influence on the active dispersion of *Ae. aegypti* in these properties.

## METHODS

### Location of Study

**Figure 1:**
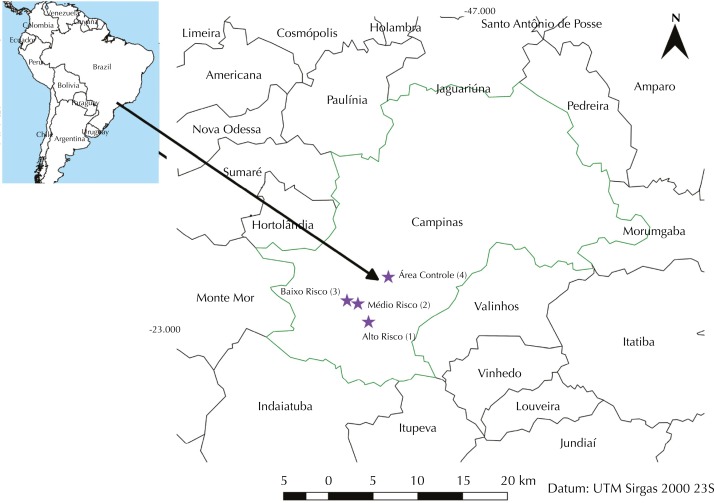
Location of study areas in the municipality of Campinas, state of São Paulo, Brazil.

This study was conducted in the municipality of Campinas, in the state of São Paulo, Brazil, located 22°57' South latitude and 47°07' West longitude, infested by *Ae. aegypti* since 1991^a^. Campinas is 98.9 km from the capital of São Paulo state and had a population of 1,142,620 inhabitants in 2016 (Figure 1). In the period from 2010 to 2016, the municipality registered approximately 120,000 cases of dengue, being classified by the Brazilian Ministry of Health as a priority due to its geographical location and incidence of infection. It is connected by several roads with intense flow of vehicles, in addition to three airports, being one international and two state airports. It comprises the headquarters of three universities, a large industrial pole, and a varied shopping center. Such circumstances lead to an intense flow and circulation of people, increasing the possibility of transmission of arboviruses and spreading them to other areas of the state and the country. From 2010 until early 2017, Campinas was responsible for about 10% of cases of dengue in the state of São Paulo^b^.

### Strategic Points

Consist in properties, usually not residential, registered by municipalities according to some criteria, such as business, existing containers, turnover of containers, and adherence to proper care by those responsible, being classified according to risk of infestation as high, moderate, and low^9^.

### Selection of Areas and Study Period

**Figure 2:**
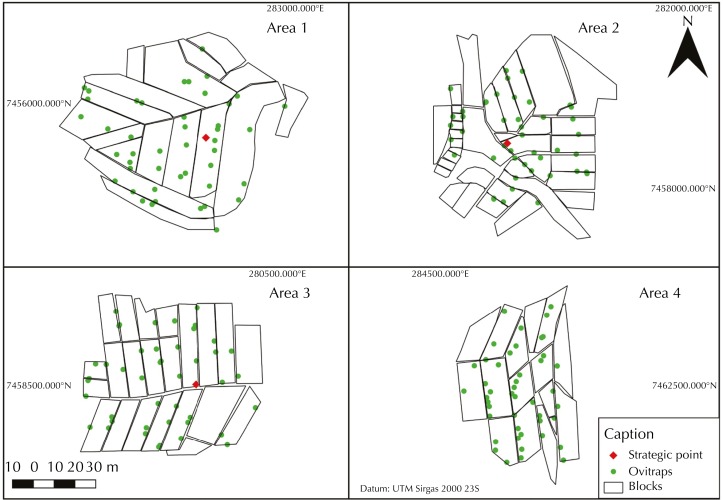
Spatial location of installed ovitraps according to areas of strategic points; spatial location of the blocks analyzed in the fields; location of strategic points, at the center of each area of high, moderate, and low risk. Campinas, state of São Paulo, Brazil, 2015–2016.

We selected four study areas (Figure 2), three of them with presence of SP (areas 1, 2, and 3) and one control area (area 4) without SP. The selection considered the risk classification of the SP. These areas were studied for 12 months, from October 2015 to September 2016, period chosen because of the increased levels of infestation, which typically occurs in October^7^.

### Installation of Traps

Based on the study conducted by Freitas and Lourenço^10^, who found an average dispersion of females of *Ae. aegypti* of 288 meters, oviposition traps (ovitraps)^11^ were installed in a 300-meter radius from each SP and from the center of the control area to assess the vector's dispersion.

### Follow-up Study

In SP, actions were monthly performed based on the technical standard^9^ according to the risk classification and collected larvae and pupae of *Ae. aegypti*. Traps were installed once a month, for a period of four days of exposure.

Properties were intentionally selected for installing the ovitraps, considering the distance of the SP and guaranteeing the distribution in all directions. In the four areas 180 ovitraps were installed, 45 in each of them. To avoid possible influence on the results, no other SP was considered in addition to those studied at less than 500 meters from the edge of the area.

We also carried out follow-up activities of the larval infestation in all properties of the selected areas. The period to visit all the buildings of the area accounted for three months, defined as visiting cycles, with four repetitions during the study – i.e., we completed four cycles. To ensure greater homogeneity, each month a third of each area was visited, simultaneously concluding the coverage of the four areas, thus preventing the seasonality of the vector to interfere with the evaluation of the results. During the activity, we recorded and classified all the containers that constituted potential breeding sites for *Ae. aegypti*, in addition to those in which we found larvae or pupae, which were collected and identified in the laboratory.

The positivity and amount of containers with larvae or pupae in each studied SP were monthly recorded. The same frequency was used for estimating the density of eggs and positivity of each ovitrap of the study area. In residential buildings, the amount of existing containers and positive for larvae or pupae of *Ae. aegypti* was registered, in addition to BII, which is the relationship between number of buildings with larvae or pupae of mosquitoes and the number of researched properties. All data were grouped in cycles of three months for analysis.

We may verify if the dispersion of *Ae. aegypti* occurs from each SP by investigating if clusters with excess of eggs or vector larvae or pupae containers are formed, respectively, in ovitraps and in the buildings closer to the SP. To test this hypothesis, we used the *G*_i_ spatial statistic, proposed by Getis and Ord^12^ ^,^ ^13^, which detects spatial agglomeration around the localities of interest^14^, i.e., the properties deemed SP.

Hence, *G*_i_ can be defined as:

$$G_{i}=\frac{\Sigma_{j}w_{ij}(d)x_{j}}{\Sigma_{j}x_{j}}para\;i\#j,$$

where *w_ij_*(*d*) is a symmetric spatial weight matrix (0-1), being 1 to all points within a distance *d* of a given point *i* (SP), and 0 for all points out of that distance; whereas *x_j_* is the measure of interest (number of eggs or larvae or pupae containers of *Ae. aegypti*) at the point *j* (property with egg traps or collection of immature forms).

The *G*_i_ statistic features a direct interpretation on how the data are distributed in space. Significantly high values of *G*_i_ points to the existence of high rates of occurrence of this attribute, being the opposite an indication of a low-value group.

Analyses were performed in the “spdep” package^15^ of the R program^16^, and their results were considered significant for p values lower than 5%.

This study was submitted for approval by the Ethics Committee in *Plataforma Brasil* [Brazil Platform System], according to presentation certificate for ethical evaluation no. 43813015.9.0000.0059, and approved according to opinion no. 1,082,780.

## RESULTS

### Positivity of SP, Ovitraps, and BII

**Figure 3:**
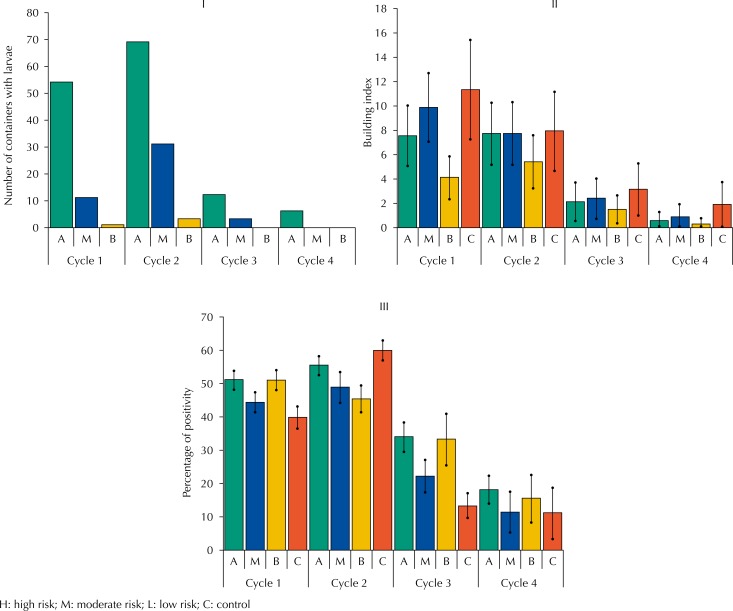
(I) Number of containers with larvae and pupae of Aedes aegypti in strategic points, according to visiting cycle and risk classification area. (II) Building infestation Index, according to visiting cycle and risk classification area. (III) Percentage of ovitrap positivity, according to visiting cycle and risk classification area. Campinas, state of São Paulo, Brazil, 2015–2016.

SP of high-risk areas, as expected, featured the highest number of containers with larvae or pupae of *Ae. aegypti* than the SP of moderate and lower risk in all visiting cycles (Figure 3, I). The largest amounts of positive containers in all areas were found in the first two cycles.

During the same period, we surveyed 5,673 properties to verify the presence of potential breeding sites for larvae and pupae of *Ae. aegypti* and positivity, which allowed us obtaining the BII for the four areas of study in the four cycles of quarterly visits. In the first two cycles, we observed higher values than in the 3^rd^ and 4^th^ cycles. We observed the highest and lowest values of the indicator, respectively, in the control area and in the area with presence of low-risk SP, in all visiting cycles (Figure 3-II).

In Figure 3-III we present ovitrap positivity per study area and visiting cycle between October 2015 and September 2016. In the first two cycles, positivity proved to be independent of the existence of SP in the areas. Positivity decreased in cycle 3, reaching the lowest values in cycle 4. In these two cycles, the high- and low-risk areas featured the highest positivity.

The direct relationship between SP positivity and risk in the area was not evidenced in the assessment of BII and ovitrap positivity. Furthermore, the control area (without SP) featured the highest BII in all cycles and highest ovitrap positivity in cycle 2 (Figure 3). However, the amount of positive containers in the SP, BII, and ovitrap positivity were consistent over time, showing similar seasonal behavior. These three indicators featured the highest values in cycles 1 and 2, intermediate values in cycle 3, and the smallest values in cycle 4.

### *G*_i_ Statistics

**Figure 4:**
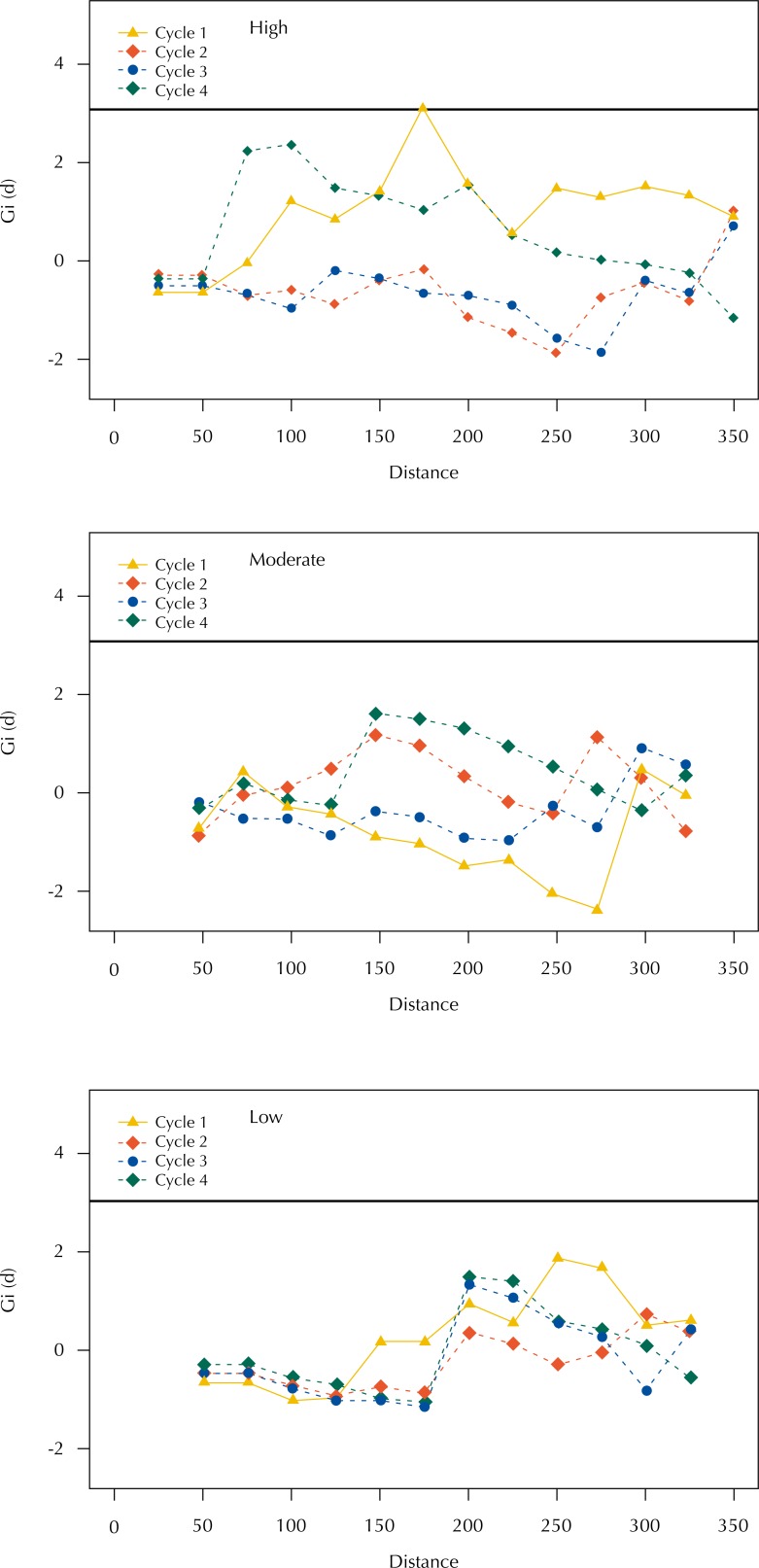
Relationship between positive traps and location of strategic points, according to visiting cycle and *Gi* statistics for areas of high, moderate, and low risk. Campinas, state of São Paulo, Brazil, 2015–2016.

**Figure 5:**
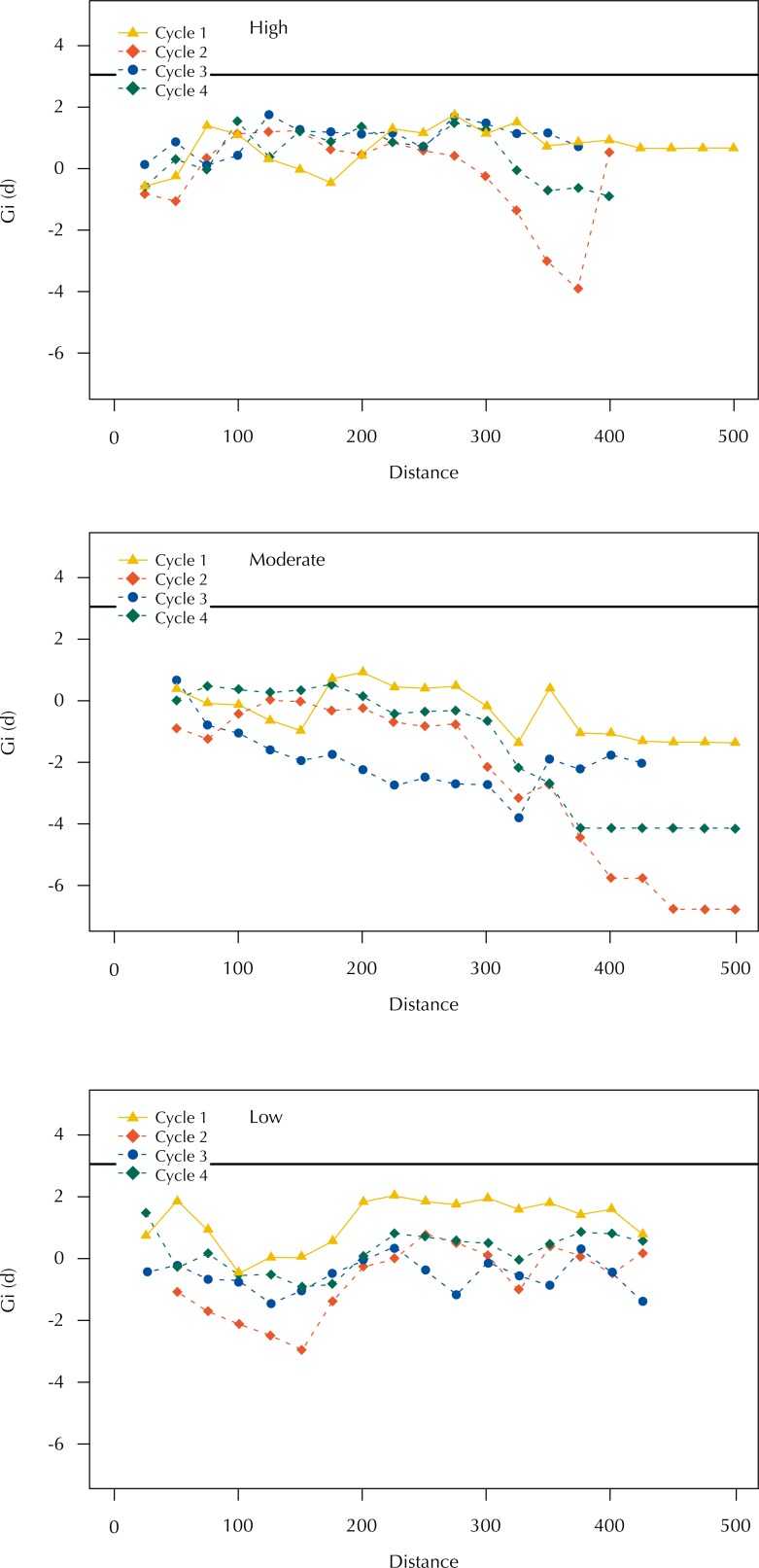
Relationship between positive buildings and location of strategic points, according to visiting cycle and *Gi* statistics for areas of high, moderate, and low risk. Campinas, state of São Paulo, Brazil, 2015–2016.

In Figures 4 and 5 we present the results of the application of the *G_i_* statistics based, respectively, on the numbers of eggs collected in the ovitraps and on the number of positive containers found in the buildings, considering the distances of ovitraps and properties of SP, for each of the four visiting cycles. In all situations, with exception of the high-risk area in cycle 1 (with agglomeration of high values of number of eggs at the 175-m distance around the SP), we did not find high-value clusters. Both the number of eggs collected in the ovitraps and the number of positive containers found in the buildings presented random distribution in relation to the SP location, and we may state such is not associated with the presence of SP.

## DISCUSSION

Reintroduction of *Ae. aegypti* into the state of São Paulo in the 1980s was detected by the SP, which at that time consisted in an effective indicator of entomological surveillance, with monitoring by the use of larvae traps. The passive vector's dispersion, mainly found in tires, began by the intense trade of this product between the municipalities of regions where the first outbreaks were detected, spreading to the rest of the state^8^.

Between 1985, the year when *Ae. aegypti* was detected in the state of São Paulo, and 1988, authors of a study on the region of São José do Rio Preto, in the Northwest of the state, showed that the vector was firstly detected in SP and then in properties from neighboring areas. This situation was identified by delimitations of focus, which consisted in expanding the area towards the vector, seeking possible outbreaks of *Ae. aegypti* in the neighborhood, in such a way the role of SP was characterized as responsible for its dispersion^8^.

Henceforth, surveillance and vector control activities in SP are indicated by the PNCD^5^, aiming both at containing the infestation in these buildings (considering the large supply of containers) and at avoiding them to be dispersed to neighboring buildings. Basically, the fortnightly visit to SP is recommended, in addition to the inspection of larval positivity, and chemical treatment whenever deemed necessary^9^.

In our study we show that SP continue to present important positivity, indicating a possible limitation of the technique currently used, which needs to be reviewed. Regarding its performance as disperser for buildings in its surroundings, the *G*_i_ statistics demonstrated that the infestation of the evaluated areas is independent of the SP infestation.

Surveillance and control activities of SP are basically the responsibility of the municipalities and face a series of difficulties, such as failed periodicity because of lack of field-work staff, noninvolvement of the owners, lack of effective actions of health surveillance, and not administering chemical treatment when necessary – either by technical problems, such as lack of equipment maintenance, or operational failure. Considering these difficulties and our results, agencies responsible for the standardization of surveillance and control activities should reformulate such actions, considering the importance of SP, but without deeming them responsible for the vector dispersion in neighboring properties.

Hence, the evaluation of SP becomes more important from the point of view of health surveillance than from the *Ae. Aegypti* control program's itself. One option would be to increase the intervals between the visits of control agencies to these buildings in periods without transmission of arboviruses, thus requiring smaller operating and economic effort. Reducing the number of mosquitoes is always a priority of the program, which must adapt the strategy to a more sustainable operation, less expensive, and of greater resolution regarding the reduction of the infestation, mainly with the involvement of those responsible for the properties.

In a study conducted in Sri Lanka, Louis et al.^17^ showed that positivity in nonresidential buildings with a large number of containers, such as SP, is higher than that found in residential properties, and they concluded that vector control strategies must be expanded to these locations, as it is done nowadays by the PNCD^5^ in Brazil. These findings are similar to ours, as well as to the study conducted by Barbosa et al.^7^

The results of Getis et al.^16^ in Iquitos, Peru, showed that the increase in the number of containers in a property increases the risk of infestation by *Ae. aegypti*, and the clusters of positive containers in households change over time. For the authors, the infestation of a house results from the inhabitants' practices for handling the containers and from the ecology of the *Ae. aegypti* behavior when egg laying. Our results corroborate these previous studies, and we may infer that the existence of SP with high supply of containers does not interfere with the positivity of properties in their surroundings. This indicates that the role of SP has changed, which is no longer the propagator of the vector.

Concerning other entomological measures considered in our study, we may address indicators based on ovitraps and the building index. The former are notoriously sensitive to indicate the presence of the vector in low density, but tend to have high positivity in situations with persistent infestation established for a long time, as we could observe in the study area, with positivity values around 50% in the first two visiting cycles. Therefore, such indicators have a reduced power to discriminate areas with higher or lower levels of infestation. On the other hand, the BII allows evaluating the vector infestation and the distribution and classification of containers, information that can be used by managers to develop action strategies, but such has higher operating costs.

Similar seasonal behavior between the representative indicators of infestation in SP, the oviposition traps, and in the buildings showed that, although there is no longer a spatial relationship between the first and the latter, the temporal relationship persists, as already demonstrated in a study by Barbosa et al.^7^ Thus, a stability of infestation is suggested in the area, considering the similarity of the indicators in the several visiting cycles. This is a result that could be considered in the reformulation of the role of SP, in such a way to use information generated by them for creating an entomological indicator. Considering the small number of SP in relation to the total amount of properties in their surroundings, an entomological indicator based on SP positivity would have lower operating costs than the building index. We must question if this indicator would have greater sensitivity than oviposition traps or sensitivity similar to the BII for differentiating areas according to level of infestation.

As possible limitations of our study, we mainly highlight the amount of buildings that were found closed at the time of domiciliary visits, which impaired the evaluation of the infestation as measured by BII. Another issue could be the quarterly measure instead of the monthly measure of BII because of operational-capacity limitations. The first issue was circumvented with the outline of a sample size suitable to cover losses, and the second, with the establishment of quarterly groupings according to the seasonal behavior of the vector. We emphasize the use of *G_i_* spatial statistics to test the hypothesis of the study, which brought an important methodological contribution. To illustrate the use of this tool, we mention the studies conducted by Kracalick et al.^18^ for analyzing the anthrax standard in Kazakhstan; by Khormi et al.^19^ to assess clusters of cases of dengue on a monthly basis in the districts of Jeddah, in Saudi Arabia; and by Bhunia et al.^20^ to assess clusters of Kala azar in the Vaishali district in India.

Our hypothesis was partially rejected, because we cannot state that SP are responsible for the vector's dispersion in properties within their neighborhood. We expected to identify clusters with high amount of eggs and positive containers at distances close to the SP, which did not occur. On the contrary, spatial distributions of these two variables were random in relation to the SP location. Furthermore, the fact that the control area, without presence of SP, did not feature positivity lower than the other areas may be an evidence of the nonnecessity of a propagator for handling the infestation. Thus, we highlight the importance of reviewing the strategy adopted for SP, with more sustainable actions, seeking balance from the technical, operational, and economic point of view, since it is a high-cost activity. An important issue is the improvement of sanitary conditions of the buildings, promoting different actions for the handling of containers. We must consider the geographic location for evaluating the risk of spread of arboviruses, since these buildings are major producers of mosquitoes.

## Footnotes

a Secretaria de Saúde do Estado de São Paulo, Superintendência do Controle de Endemias. São Paulo: Sucen; 2017. Unpublished internal data.

b Department of Health of the State of São, “Prof Alexandre Vranjac” Epidemiological Monitoring Center. São Paulo: CVE; 2017. Available from: http://portal.saude.sp.gov.br/cve-centro-de-vigilancia-epidemiologica-prof.-alexandre-vranjac/zoonoses/dengue/dados-estatisticos
